# Early rehabilitation of cancer patients – a randomized controlled intervention study

**DOI:** 10.1186/1471-2407-13-9

**Published:** 2013-01-07

**Authors:** Cecilia Arving, Inger Thormodsen, Guri Brekke, Olav Mella, Sveinung Berntsen, Karin Nordin

**Affiliations:** 1 Department of Oncology and Medical Physics, Haukeland University Hospital, Bergen, Norway; 2 Department of Public Health and Caring Sciences, Uppsala University, Box 564, Uppsala SE-751 22, Sweden; 3 Department of Public Health and Primary Health Care, University of Bergen, Bergen, Norway; 4 Department of Public Health, Sport and Nutrition, Faculty of Health and Sport Sciences, University of Agder, Kristiansand, Norway

**Keywords:** Adjuvant/curative therapy, RCT with stepped-care approach, Individual stress management, Impact of event scale, Physical activity

## Abstract

**Background:**

Faced with a life-threatening illness, such as cancer, many patients develop stress symptoms, i.e. avoidance behaviour, intrusive thoughts and worry. Stress management interventions have proven to be effective; however, they are mostly performed in group settings and it is commonly breast cancer patients who are studied. We hereby present the design of a randomized controlled trial (RCT) evaluating the effectiveness and cost-effectiveness of an individual stress-management intervention with a stepped-care approach in several cancer diagnoses.

**Method:**

Patients (≥ 18 years) with a recent diagnosis of breast cancer, colorectal cancer, lymphoma, prostate cancer or testicle cancer and scheduled for adjuvant/curative oncology treatment, will consecutively be included in the study. In this prospective longitudinal intervention study with a stepped-care approach, patients will be randomized to control, treatment as usual, or an individual stress-management intervention in two steps. The first step is a low-intensity stress-management intervention, given to all patients randomized to intervention. Patients who continue to report stress symptoms after the first step will thereafter be given more intensive treatment at the second step of the programme. In the intervention patients will also be motivated to be physically active. Avoidance and intrusion are the primary outcomes. According to the power analyses, 300 patients are planned to be included in the study and will be followed for two years. Other outcomes are physical activity level, sleep duration and quality recorded objectively, and anxiety, depression, quality of life, fatigue, stress in daily living, and patient satisfaction assessed using valid and standardized psychometric tested questionnaires. Utilization of hospital services will be derived from the computerized patient administration systems used by the hospital. The cost-effectiveness of the intervention will be evaluated through a cost-utility analysis.

**Discussion:**

This RCT will provide empirical evidence of whether an individually administered stress-management programme in two steps can decrease stress as well as maintain or enhance patients’ physical activity level, quality of life and psychological well-being. Further, this RCT, with a stepped-care approach, will provide knowledge regarding the cost-effectiveness of an individually administered stress-management programme whose aim is to help and support individual patients at the right level of care.

**Trial registration:**

ClinicalTrials.gov Identifier: NCT 01588262.

## Background

Faced with a life-threatening illness, such as cancer, many patients develop stress symptoms, i.e. avoidance behaviour, intrusive thoughts and worry [1]. These are all common symptoms of post-traumatic stress disorder (PTSD), which is defined in the Diagnostic and Statistical Manual of Mental Disorders - Fourth edition (DSM-IV) [2] as a phobic and anxious reaction in the wake of a traumatic experience. Life-threatening illnesses were included as a potential traumatic event in 1994, and since then PTSD has been registered as a secondary diagnosis in many cancer patients [2]. Horowitz and colleagues [3] define avoidance as a constrict of ideas, denial of the event (both its meaning and its consequences) and emotional numbing, while intrusion includes unbidden thoughts and images, troubled dreams, repetitive behaviour and waves of feelings. PTSD has been investigated in breast cancer patients [4,5], but little is known about its prevalence among other cancer diagnoses [6,7]. A recent study from Sweden reported that, compared to age-matched controls, men diagnosed with prostate cancer had an increased risk of psychiatric treatment for depression, PTSD and use of antidepressants, regardless of risk group and treatment strategy [8]. In another study, Smith and colleagues [9] concluded that the impact of a cancer diagnosis and treatment persists over the years for many cancer survivors. Therefore, early identification of those at risk could enable treatment to minimize PTSD symptomatology.

Nordin and Glimelius [10] reported that the early identification of those in need of psychological support at a later stage of treatment is possible through screening for clinical levels of worry or depression in combination with intrusive thoughts in individuals with cancer. Further, it has been reported [11] that the effects of cancer treatment on an individual’s quality of life could be predicted using measures of avoidance behaviour. Impaired general health as well as deterioration of both physical and social functioning were correlated with extensive avoidance behaviour at the start of treatment. Since the research has been quite static regarding how to properly select, through screening, the individual patient in order to help and support him/her at the right level of care, there is a need to expand the body of knowledge [12,13].

For patients in states of intense stress, the occurrence of PTSD can be reduced if cognitive behaviour therapy (CBT) is provided one to three months after a traumatic event [14]. Also, methods based on CBT have proven to be able to improve health-related quality of life (HRQoL), reduce psychosocial stress and increase perceived personal control of treatment side-effects and disease symptoms in cancer patients [15,16]. However, at present there is a lack of solid research findings for comparisons of cost-effectiveness and outcome [14].

In the “Breast Cancer and Stress” project (BAS project) [17], the research group wanted to evaluate the effectiveness and cost-effectiveness of a stress-management intervention for breast cancer patients about to start adjuvant therapy. Methods based on CBT were used and the intervention was administered in two steps. In the first step, all participants were offered a low-intensity intervention. Thereafter, patients were screened regarding clinically relevant levels of avoidance, intrusion, anxiety and depression, and those who reported clinically relevant levels were offered more intensive treatment in Step 2 of the programme. Patients who consented to participate in Step 2 were randomized to a more intensive stress-management intervention, either in a group setting or individually. The hypothesis was that half of the individuals assigned to a low-intensity intervention would have significantly improved after treatment. For individuals who continue to have symptoms after low-intensity treatment, it is hypothesized that continued treatment in a group setting with high-intensity interventions will be more cost-effective than individual treatment.

However, since the BAS project was planned and carried through, several reports have been published describing that interventions maintaining or enhancing patients’ physical activity level were successful in decreasing stress and increasing quality of life as well as psychological well-being [18]. Anti-depressant effects have also been reported to be caused by exercise in self-referred cancer patients undergoing chemotherapy [19]. Level of physical activity as well as sleep hours relate to psychological well-being and quality of life in cancer patients [20]. Therefore, we wanted to study whether the stress management offered in the BAS project could be improved using a supplement whereby the counsellors performing the intervention specifically worked with motivating the participants to maintain or increase their physical activity level during and after adjuvant/curative treatment. Further, since there is a lack of knowledge regarding individual stress-management interventions in diagnoses other than breast cancer we decided to include various cancer diagnoses, as well as both men and women. In addition, since studies reporting on the cost-effectiveness of stress-management interventions by means of cost-utility analysis (CUA) are scarce, the interventions in the stepped-care approach will be evaluated using CUA.

This paper presents the design of a randomized controlled trial evaluating the psychosocial effects and cost-effectiveness of an individual stress-management intervention, using a stepped-care approach, for patients with various cancer diagnoses. The intervention is based on CBT, and includes encouragement to be physically active. The hypothesis is that a low-intensity intervention will be sufficient for half of the individuals to maintain or improve their psychological well-being after treatment. For individuals who continue to have symptoms after low-intensity treatment, it is hypothesized that continued treatment with high-intensity interventions will be more cost-effective than that of the control group, which entails treatment as usual.

Specific research questions are:

• Are there any differences between patients who received an intervention based on CBT and those who did not, as regards intrusive thoughts, avoidance behaviour, anxiety, depression, sleep duration and quality, and/or moderate-vigorous-intensity physical activity level?

• Is a stress intervention administered at a low or intensive level a cost-effective way to support patients with cancer?

• Is it possible to predict at baseline which patients are in need of a shorter (low level, Step 1) or longer (intensive level, Step 2) intervention?

## Method

### Patients

Patients (n = 300) over the age of 18 years, with a recent diagnosis of breast cancer, colorectal cancer, lymphoma, prostate cancer or testicle cancer and scheduled for adjuvant and/or curative treatment in the form of chemotherapy, radiation therapy and/or hormonal therapy at the Department of Oncology and Medical Physics, Haukeland University Hospital (Department of Oncology), Bergen, will be consecutively included in the present study. Criteria for exclusion are an ongoing psychiatric condition or language deficiencies in Norwegian.

### Design

**Figure 1:**
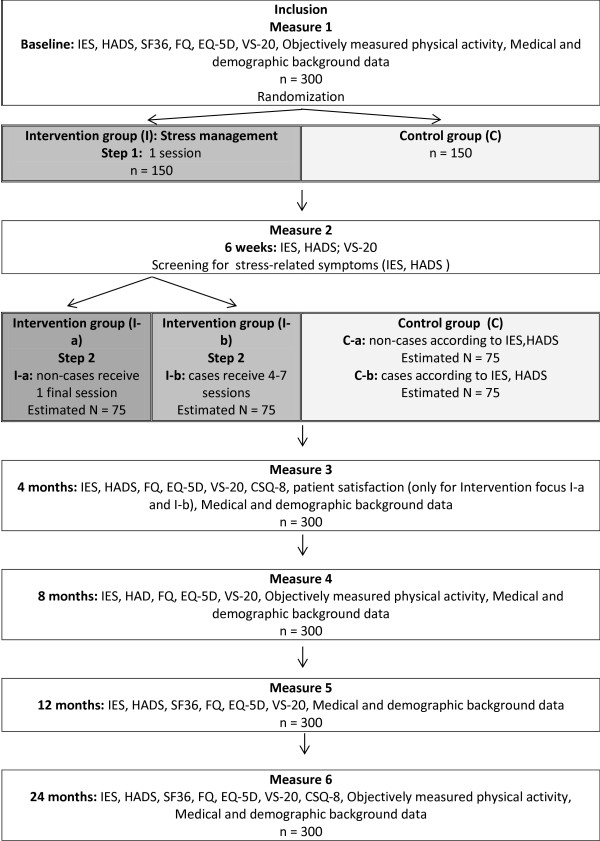
The Study design.

The study is a prospective, longitudinal intervention study with a stepped-care approach whereby patients will be randomized to individual stress-management intervention (I) or control (C), treatment as usual. A stepped-care treatment model seeks to treat patients at the lowest appropriate level in the first instance, only 'stepping up' to intensive/specialist services if clinically required. One of the key concepts in the stepped-care model is that treatment methods of varying intensities are matched to the needs of the individual patient [21]. In Step 1, all patients who are randomized to individual stress management will be given a low-intensity treatment (I-a). Those patients who do not improve after this treatment will be given a more intensive treatment (I-b) in Step 2. This makes the intervention more cost-effective, since the intensity of the treatment is adjusted to meet the needs of the individual patient. For a more detailed overview of the design, see Figure 1.

### Interventions

In the individual stress-management intervention the sessions will be 45-60 minutes long, and will take place at the Department of Oncology or be conducted by telephone if the patient has been discharged from the hospital. If patients live at a great distance from the hospital some parts of the intervention may be conducted by telephone, which has been shown to have a similar effect [22]. The individual stress-management intervention is planned to be completed within six-months of inclusion.

#### Step 1 (I-a)

All patients will receive one counselling session when they start adjuvant/curative therapy at the Department of Oncology, and a follow-up session face-to-face or over the telephone. At the first session a thorough evaluation of the patient’s psychosocial status will be conducted, according to a model used in a previous study [23]. At the first session patients will receive written information about causes and symptoms of stress, self-care measures to influence stress such as a daily registration of events and behaviour, scheduled behavioural and physical exercises, along with a short relaxation training. Patients who do not report clinically significant levels of stress at the six-week assessment will only participate in these two sessions. They will, however, be evaluated regularly for two years (see Figure 1).

#### Step 2 (I-b)

Patients who report clinically significant levels of stress, such as intrusive thoughts/avoidance behaviour (measured using the Impact of Event Scale (IES) [3]) and/or worry and depression (measured using the Hospital Anxiety and Depression Scale (HADS) [24]) at the six-week assessment will be included in Step 2. Step 2 includes more intensive stress management, with the remaining four to seven sessions devoted to the themes “What is stress?”, “What are the symptoms of stress?” and “Irritability, relationships, the body and pleasure, as well as an orientation towards the future”. These topics are based on an earlier intervention [25]. At all sessions the counsellor will motivate the patient to maintain or increase his or her physical activity level during and after the cancer treatment. At the final session, the counsellor will evaluate the patient’s use of the material and ability to manage his or her stress reactions.

#### Control group (C)

This condition includes the care offered to all patients, e.g. all study participants and non-study participants at the Department of Oncology. It consists of regular contact with the patient’s own doctor and hospital staff, as well as the opportunity to take part in the common rehabilitation programme, including patient education and physical training.

### Recruitment

Patients with a previously listed cancer diagnosis will be invited as soon as a decision has been made to initiate treatment for cancer. Eligible patients will receive written information about the study by post, informing them that they will be contacted by telephone within a week by a member of the project staff. During this telephone call the patients will have the opportunity to ask questions and receive oral information about the study, before they decide whether or not they would like to participate. If they agree to participate they will receive a questionnaire by post containing a request for a written informed consent and baseline questionnaires. A physical activity monitor will be mailed to participants when the baseline questionnaires have been returned (Measure 1). Thereafter patients will be randomized to one of the two conditions, intervention or control. In consultation with each patient who is randomized to the individual stress-management intervention (I), an appointment for the first session will be decided. Individual stress-management treatment will be conducted by specially trained staff at the Department of Oncology, who will schedule appointments after consultation with the patient. Staff members conducting the treatment will receive ongoing supervision throughout the project by a senior researcher.

### Data collection

Data will be collected using valid, psychometrically tested and standardized self-reported questionnaires. Primary outcome (intrusion and avoidance) will be measured using the Impact of Event Scale (IES) [3]. Secondary outcomes regarding psychological well-being will be collected using the Hospital and Depression Scale (HADS) [24], “Daily Stress” (VS-20) [26], Short Form Health Survey (SF-36) [27] and Chalder’s Fatigue Questionnaire (FQ) [28].

Physical activity level as well as sleep duration and -quality will be recorded using the SenseWear™ Pro_3_ Armband (Armband (BodyMedia Inc., Pittsburgh, PA, USA), according to manufacturer instructions, for seven consecutive days [29]. The SenseWear™ Pro_3_ Armband is an activity monitor that is easily applicable, estimates energy expenditure from five (both physiological and mechanical) outputs, and has been found to be valid during free-living activities [30] as well as in groups of cancer patients [31].

The generic Euro Quality of Life 5-Dimensional Classification (EQ-5D) [32] will be used assess health status, and is suitable for health economic analyses. Data from medical records will be used to obtain patients’ demographic and medical background, and information on the utilization of hospital services will be collected from the hospitals’ computer systems/medical records. Information on the utilization of other professional care facilities will be collected from patients’ self-reports. Information regarding sick leave will be collected through self-reports. Attendance at the individual sessions will be recorded by the counsellor.

In addition, the intervention as well as the treatment programmes offered through the regular services of the Department of Oncology will be evaluated using the Client Satisfaction Questionnaire (CSQ-8) [33].

All questionnaires will be sent to the patients’ homes together with a stamped, pre-addressed envelope. Patients who have not returned the completed questionnaires after 14 days will be contacted by telephone by a member of the project staff to ensure as high a response rate as possible.

### End-points and statistical analysis

Power calculations have been done for the IES based on data from another study [23]. On the basis of these conditions (power = 0.8, p = 0.05 and effect size = 0.59), at least 128 patients must be included in each group to detect a significant difference on the IES. To account for the expected dropout and loss of follow-up rates of 15%, we intend to include 300 patients. ANOVA will be used to analyse differences between groups and for repeated measures over time of the continuous variables, while nominal (categorical) variables will be tested using chi square (exact). Although all the criteria for normal distribution are not met with this selection, the parametric tests are sufficiently robust to be used. Patients’ levels of stress-related symptoms will be categorized according to standardized cut-off points. A hierarchical linear regression analysis will be used to examine which variables predict stress symptoms and the utilization of health care services, as well as sick leave and return to work. Net values of the total health care costs will be calculated for the intervention and control groups. Cost–utility analysis (CUA) using quality adjusted life years (QALYs) is a general approach in health economic evaluation [34,35]. QALY is an overall measure of health outcome that weighs a patient’s life expectancy against an estimate of his or her HRQoL score (measured on a 0–1 scale). Cost-effectiveness ratios (CERs) will be calculated as (Δ intervention costs + Δ health care costs)/ Δ QALYs for intervention compared with control. The number of QALYs gained/lost relative to baseline will be calculated for each patient assigned to the intervention or control group as the area under the baseline-adjusted utility curve.

### Ethical considerations

The study has been approved by the Medical Research Ethics Committee and the Data Inspectorate of Norway (2010/1911). The intervention in the project will be administered by specially trained staff, and will be supervised continuously so that any potentially harmful effects can be detected early in treatment and corrected immediately. Since the design includes a control group who will receive treatment as usual, it will be possible to monitor patients for potentially harmful effects. The questionnaires used in the present study have been chosen with care, in terms of both length and number. Recording physical activity level and sleep using an objective monitor as in the present study has been reported to be feasible and acceptable to cancer patients, and compliance has been reported as high [29].

## Discussion

By screening for intrusion, avoidance, anxiety and/or depression, the intervention at low- or high-intensity level will hopefully help and support the individual patients, both men and women with various cancer diagnoses, at the right level of care. In the present study, using a stepped-care approach, it will be possible to evaluate the effectiveness of both a low-intensity general stress-management intervention and a high-intensity individually tailored stress-management intervention compared to standard care. Further, we will gain knowledge concerning whether the stress-management intervention administered here can motivate the participants to maintain or increase their physical activity level during and after adjuvant/curative treatment. Health care and society stand to gain substantially from the planned intervention programme, in the form of both individualized psychosocial support and increased psychological well-being. This may result in a reduced utilization of health care, preserved work capacity or a quicker return to work. The high-intensity individually tailored support will only be offered to patients who show a clear need, which may be an economically beneficial model, for both the patient and society. For research purposes, it is also important to gain increased knowledge about how to select and deliver effective supportive intervention for patients with various psychological needs and cancer diagnoses. The present study will be integrated within the usual care at the Department of Oncology. This can facilitate the implementation of evidenced-based psychosocial care for cancer patients.

The project has limitations, which should also be noted. Most stress-management interventions have been delivered in group format. The lack of a third group, undergoing the stress-management intervention in a group setting, makes it impossible to compare the effects of different stress-management intervention settings (individual vs. group) on stress symptoms compared to a control group. However, if a third randomization group had been added to the project it would have prolonged the time frame by approximately 12 months, which was not economically justified.

## Competing interests

The authors declare that they have no competing interests.

## Authors’ contributions

CA, IT, GB, OM, SB and KN were involved in the design of the study. KN is the principal investigator of this study. CA will be responsible for the supervision of the interventions. IT will be responsible for data collection and will, together with CA, GB, GK, SB and KN, conduct the analysis and interpretation. All authors have read and approved the final version of the manuscript.

## Pre-publication history

The pre-publication history for this paper can be accessed here:

http://www.biomedcentral.com/1471-2407/13/9/prepub
